# PubMed: an untapped source for open educational resource images

**DOI:** 10.5195/jmla.2026.2210

**Published:** 2026-04-01

**Authors:** Ellie Svoboda, Teresa Connolly

**Affiliations:** 1 ellie.svoboda@cuanschutz.edu, Strauss Health Sciences Library, University of Colorado Anschutz, Aurora, CO; 2 teresa.connolly@cuanschutz.edu, College of Nursing, University of Colorado Anschutz, Aurora, CO

**Keywords:** PubMed, open educational resources, OER, images, repositories

## Abstract

**Background:**

Open Educational Resources (OER) are free learning materials that benefit students in higher education, including in the health sciences. As more health sciences OER materials are created, there is a need for openly licensed health sciences images. Traditional OER repositories lack specialized health sciences imagery while PubMed is a biomedical database that has potential to fill this gap.

**Case Presentation:**

A nursing faculty partnered with a health sciences librarian to search PubMed for openly licensed images for a pathophysiology OER textbook. The librarian used existing filters in PubMed to identify articles that have Creative Commons licenses as well as images. The nursing faculty assessed these images and added relevant ones to the pathophysiology textbook.

**Conclusions:**

PubMed is a free resource that health sciences librarians use on a regular basis. Utilizing the database to find openly licensed materials allows librarians to use a familiar tool for a new and exciting purpose.

## BACKGROUND

Open educational resources (OER) are defined as “learning, teaching and research materials in any format and medium that reside in the public domain or are under copyright that have been released under an open license, that permit no-cost access, re-use, re-purpose, adaptation and redistribution by others” [1]. Textbooks are one type of OER that are created and implemented within higher education. The process of eliminating a textbook from a course and replacing it with an OER has positive benefits in higher education such as maintaining final course grades and preventing course withdrawals [2]. Furthermore, when an OER textbook is instituted within a course there is a reduced financial textbook burden for students and this can disproportionately affect students of diverse backgrounds by reducing their anxiety [2–4].

Despite these known benefits of OER, adoption within health sciences educational programs such as nursing remains challenging [5]. Luo [6] completed a systematic literature review of barriers to the integration of OER and found that discoverability of OER was a consistent problem. Besides difficulty finding materials, reviewing potential OER is time consuming and with fragmented repositories faculty could miss key resources. OER repositories have a variety of materials beyond textbooks, such as videos, course modules, and virtual learning environments which only complicates the discoverability of resources.

Within health sciences education, the implementation of anatomical illustrations as a visual learning tool is widely utilized. Over the years, hundreds of textbooks on medical illustrations have been created and evolved from simple hand drawings to digital photography [7]. Creating images in OER is difficult because many faculty lack funding to pay medical illustrators and most do not have illustration skills themselves. This leads faculty to search for images which can be extremely time consuming and results frequently disappoint. Anecdotally, when the authors were working with health care educators on creating OER one of the most common questions by faculty is “where do I find medical OER images.” Google Images is a common source for openly licensed images, but using it for medical images can be frustrating. For example, when one searches “heart diagram” in Google Images, hundreds of results are returned including basic renderings of the heart as well as complex labeled images. However, when the search is restricted images that have a Creative Commons license there are far fewer images many of which are more simplistic or even cartoon illustrations.

Other sources for health sciences OER images include image repositories such as the Public Health Image Library (PHIL) from the CDC, the Cell Image Library from the Center for Research in Biological Systems, and Openi from the NLM [8–10]. These resources include many relevant images; however, they are fractured into subdisciplines and require the same search to be run in multiple repositories which can be time consuming.

The following case report describes the process that a nursing faculty and health sciences librarian used to identify quality openly licensed images for an OER textbook. We will describe how we utilized PubMed, an unorthodox source for OER but a common database for medical research, to locate openly licensed health sciences illustrations. The process is easy to follow and could be replicated by other health sciences librarians.

## CASE PRESENTATION

The University of Colorado Anschutz Medical Campus is an R1 research university with six professional schools that serve 4,500 students. The University of Colorado Anschutz College of Nursing has both undergraduate and graduate programs with more than 1,100 students. The undergraduate program has roughly 500 students and College of Nursing faculty are working to lower the cost of textbooks for undergraduate nursing students. This involves adopting existing nursing OER textbooks as well as creating new OER textbooks and learning materials. One of the creation projects in this effort is authoring a textbook on undergraduate pathophysiology that will be licensed CC-BY-NC. Pathophysiology is a core nursing curriculum class and there is not yet an OER textbook that can replace the current commercial textbook used by the College. Creating an OER pathophysiology text will significantly reduce the cost burden of textbooks for CU Anschutz nursing students as well as students in nursing and health sciences programs at other institutions.

Creating a textbook is a significant undertaking that involves not only writing the content that students need to learn but also generating activities and quizzes to help students reinforce and check their own knowledge. Visual aids and images are an important component of reinforcing concepts and providing contextual information. These aid in the learning of most subjects but are especially important in pathophysiology due to the variety of disease processes, which are most effectively communicated visually.

The faculty authoring the pathophysiology text do not have graphic design or illustration skills and the medical illustrator at the university does not offer services to individual faculty. Hiring a freelance medical illustrator or graphic designer was also prohibitively expensive. Instead, the authors looked for existing, openly licensed (specifically CC-BY) images that can be incorporated into an OER text. Unfortunately, finding openly licensed images on niche healthcare topics in the usual OER repositories was proving to be challenging.

The College of Nursing has a strong relationship with the Strauss Health Sciences Library and the lead author reached out to the nursing liaison librarian for assistance.

Librarians have expertise in copyright and database searching which makes them natural partners to assist health sciences faculty with locating openly licensed materials. The nursing liaison librarian has a special interest in copyright and had previously discovered that PubMed has a filter for identifying open access (OA) articles [11]. This functionality made PubMed an exciting and untapped resource for openly licensed health sciences images.

In addition to a general filter that finds articles with any open license, PubMed also has filters that allow users to specify particular Creative Commons licenses. Below is a table of the Creative Commons licenses and their corresponding filters in PubMed [12].

**Table 1:** 

License	PubMed filter	Function
CC-BY	"pmc cc by license"[filter]	Allows for unlimited reuse as long as attribution is provided.
CC-BY-SA	"pmc cc by-sa license"[filter]	Allows for unlimited reuse as long as attribution is provided and derivative works apply the same license.
CC-BY-NC	"pmc cc by-nc license"[filter]	Allows for reuse but prohibits any commercial applications of the work. Requires attribution.
CC-BY-NC-SA	"pmc cc by-nc-sa license"[filter]	Allows for reuse but prohibits any commercial applications of the work. Requires attribution and that derivative works apply the same license.
CC-BY-ND	"pmc cc by-nd license"[filter]	Allows for unlimited sharing but prohibits any derivative works. Requires attribution. Is not compatible with OER.
CC-BY-NC-ND	No PubMed search filter for this license	Allows for sharing but prohibits any commercial use and derivative works. Requires attribution. Is not compatible with OER.

The librarian decided to use the filter for CC-BY articles ("pmc cc by license"[Filter]) because works under that license can be used in any OER context and would be compatible with the pathophysiology textbook. The librarian chose not to use the general OA filter because it retrieves articles with all of the Creative Commons licenses including the non-derivative licenses that are incompatible with OER. The librarian also used the filter for articles that are not copyrighted and in the public domain ("pmc cc0 license"[Filter]) because those articles would also be eligible to be incorporated into an OER textbook.

To find images for the pathophysiology textbook, the lead author created a list of more than fifty diseases that would be featured in the book. The librarian used this list to search in PubMed for articles on that topic. To maximize her time and to keep the result sets highly focused, the librarian searched basic search terms for the diseases along with either the field tag for titles and abstracts [tiab] or for titles [ti]. Depending on the scope of the research, these terms were combined with the word pathophysiology with the title and abstract field tag [tiab]. These results were then limited with the Creative Commons filters. From the resulting pool of articles, the librarian manually went through the first ten to thirty results and identified which articles had figures. These articles were then downloaded into an EndNote library with groups for each disease state. The EndNote library was then compressed and sent to the nursing faculty for review. The goal was to identify images that presented the classic form of a disease with clarity and the key components labeled.

When reviewing these images, the nursing faculty often found hyper-specific x-rays or photographs of unique case presentations which were mostly unsuitable for an undergraduate textbook. However, occasionally, there are simple and straightforward illustrations of the pathophysiology of a disease state. Below is an example of an ideal illustration for undergraduate nursing students that visually conveys the pathological process of carpal tunnel syndrome [13]. This image was downloaded from PubMed as a figure in a guideline on carpal tunnel syndrome [14]. The original source of the image is a site called WikiJournal of Medicine that includes many Creative Commons licensed diagrams and illustrations on health sciences topics.

The nursing faculty uploaded this image into Pressbooks, the online platform that the OER textbook is hosted within, in the chapter that covers carpal tunnel syndrome. When students are reading about the pathophysiology of carpal tunnel syndrome, this image will provide a visual experience that meets more learning styles and student needs.

**Figure 1:**
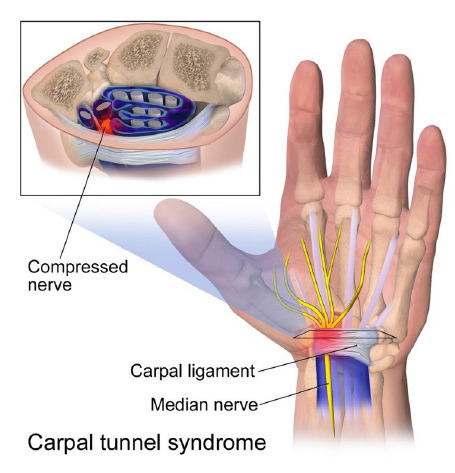
Carpal Tunnel Syndrome by BruceBlaus is licensed under a CC-BY-3.0 license.

This image is available under the Creative Commons Attributions license and permits use, distribution, and reproduction in any medium as long as attribution is provided. To provide an adequate attribution, users must include the title of the work, the author of the work, the source of the work, and the license of the original work [15].

The OER undergraduate pathophysiology textbook is still being written but so far, the faculty author has been able to add six illustrations that were identified in PubMed using the methodology above. As more chapters are authored, this number will likely increase. There are still some diseases that need images that the authors haven’t been able to locate in either PubMed or other OER image repositories.

## DISCUSSION

This methodology can be reproduced by health sciences librarians at any institution including academic campuses that are engaging in the creation of Open Educational Resources projects such as textbooks, case studies, videos, or simulations, as well as hospitals at which physicians are lecturing at society conferences and would like to include copyright-safe images in their slides. Most health sciences librarians use PubMed on a frequent basis and will be familiar with the recommended field tags.

Another benefit of finding images in this way is that librarians can discover repositories and collections of openly licensed images of which they might not otherwise have been aware. That was the case with the WikiJournal of Medicine, which the authors found when searching for carpal tunnel syndrome images. This resource has now been used and searched directly for other images for the textbook.

While this method can create many opportunities, there are also some limitations. First, an article with an open license does not guarantee that the figures included within are also openly licensed. Librarians need to provide some education to patrons who are evaluating the figures regarding the complexity of article and image licensing. This will help prevent a well-intentioned patron from using a copyrighted image by accident. If librarians would like to learn more about copyright and how Creative Commons licenses work, the content of the Creative Commons Certification Course is freely available online [16].

It is also important to clearly delineate the role of the librarian. The nursing liaison librarian did not assess the figures because she does not have that expertise. The onus for determining the appropriateness and veracity of images must lie with the faculty.

Another limitation is that most of the figures are in formats that are difficult to edit. With many Creative Commons images, authors are legally allowed to make modifications, such as moving the location of a caption or adding additional captions. This is more difficult when the image is in a format such as a PDF, which may require additional software and expertise which may not be accessible to everyone.

Finally, many of the figures are highly specific to the content of the article. There are often case reports highlighting an unusual presentation of a disease state. Generally, for a textbook, it is better to present undergraduate students with the typical or expected version of a disease or condition. However, if the audience is clinicians or residents, these specific images might be very helpful. This means that authors may have more sifting to do to find the images that fit the needs of their audience.

If health sciences librarians keep these limitations in mind, this method of searching PubMed is an easy way to help patrons find openly licensed images for their projects without having to learn the quirks of a variety of OER repositories. It has been an asset to the authors at the University of Colorado Anschutz Medical Campus and has been replicated for other sections of the pathophysiology textbook.

As creation and implementation of OER grows in health sciences education, more faculty will need to find openly licensed materials. Current mainstream methods and sources of openly licensed medical images are inadequate and alternative methods are needed. Searching PubMed with an OA filter is a creative approach to finding open-sourced images that capitalizes on health sciences librarians existing skills and knowledge.

## Data Availability

There are no data associated with this article.
